# From flat to stepped: active learning frameworks for investigating local structure at copper–water interfaces

**DOI:** 10.1039/d5cp00396b

**Published:** 2025-04-04

**Authors:** Johannes Schörghuber, Nina Bučková, Esther Heid, Georg K. H. Madsen

**Affiliations:** a Institute of Materials Chemistry, TU Wien A-1060 Vienna Austria georg.madsen@tuwien.ac.at

## Abstract

Understanding processes at solid–liquid interfaces at the atomic level is important for applications such as electrocatalysis. Here we explore the effects of different step densities on the structure of interfacial water at the copper–water interface. Utilizing spatially resolved uncertainties, we develop an active learning framework and train a machine-learning force field (MLFF) based on dispersion-corrected density functional theory data. Using molecular dynamics simulations, we investigate structural properties of water molecules in the contact layer, including density profiles, angular distributions, and 2D pair correlation functions. In accordance with previous studies, we observe the formation of two sublayers within the contact layer at the Cu(111) surface, whereas the structure on surfaces with a high step density is dominated by the undercoordinated ridge atoms. By systematically decreasing the step density, we identify the cross-over to when the behavior observed at the flat surface can be locally recovered. Using dimensionality reduction, we identify four distinct types of Cu environments at the interfaces, providing insights into analyzing less idealized surfaces with MLFFs.

## Abstract 2

Using active learning, the step-density dependence of the copper–water interface structure is investigated and relevant types of local environments at the interface identified.

## Graphical abstract

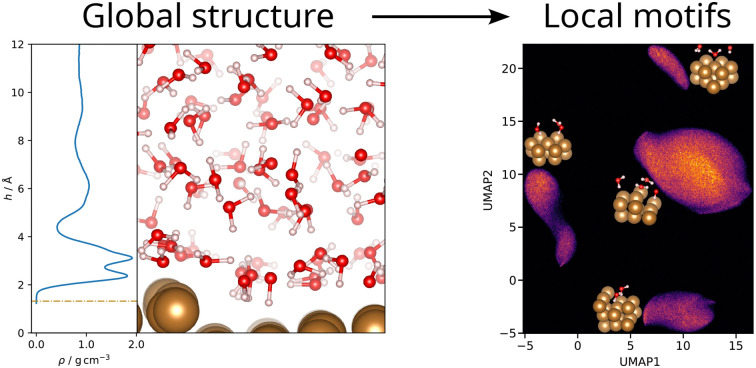

## 1 Introduction

Metal–water interfaces are of large importance in technological applications, for example in devices used in electrochemical energy conversion and storage.^1,2^ In heterogeneous catalysis not only the composition of the material, but also the surface morphology has a profound effect on performance and efficiency. In the case of electrochemical CO_2_ reduction on Cu, just changing the orientation of a single crystal already has a large impact on the selectivity toward C_2_- or C_1_-products.^3–5^ Further, the structure of both the surface and in conjunction also interfacial water has been shown to be an integral descriptor for other catalytic processes on Cu surfaces, such as the hydrogen evolution reaction and CO reduction, using *in situ* measurements at the solid–liquid interface.^6,7^

The applicability of classical force fields for explicit simulations of metal–water interfaces in molecular dynamics (MD) simulations is limited by the fundamentally different properties of the solid and liquid phases. On the other hand, the computational cost of *ab initio* calculations meant that early work was focused on clusters and ultra-thin layers of frozen water.^1,8,9^ As *ab initio* molecular dynamics (AIMD) and machine-learned force fields (MLFFs) have become available they have enabled progressively more realistic simulations of the bulk water–metal interface. These studies have established the presence of a double-peak structure in the interfacial water density for the Cu(111) surface,^10,11^ similar to observations for Pt(111) surface.^11–16^ Additionally, the distinct behavior of interfacial water on stepped surfaces has also been demonstrated for Cu–H_2_O and Pt–H_2_O interfaces.^11,14,17^ However, while global descriptors such as Miller indices and edge densities can provide insights into the bonding properties of corrugated surfaces, they can obscure the local atomistic details. Bridging the gap between morphological understanding and detailed atomic-level analysis remains a key challenge.^18^ MD simulations have historically been limited when it comes to achieving the time scales necessary to generate density profiles and analysis with local resolution. The recent rapid development of MLFFs has made it possible to obtain reliable statics for local resolution through higher-dimensional pair-correlation functions and free energy calculations.^16^ This can open up possibilities for bridging the gap between morphological insights and local atomistic understanding.

The quality of MLFF predictions is highly dependent on the data it was trained on, as the sampled structures determine the region of configuration space for which accurate predictions can be obtained. As even small representative systems for solid–liquid interfaces contain hundreds of atoms and the *ab initio* reference calculations are consequently costly, it is important to sample new configurations efficiently and only perform as many calculations as necessary. This makes it a prime task for active learning (AL).^19^ In AL procedures, new configurations are added to the training database based on the inaccuracy of the model predictions. The model is then retrained in order to improve model predictions in the region of the configuration space in which the newly selected structures lie. This procedure is iterated until a desired convergence is reached. When studying interfaces it is clearly desirable that the AL procedure can be built on preexisting data for the individual constituents,^20^ and then use an AL strategy to sample from model-based interface simulations.

In the present study, we develop a MLFF to systematically investigate the flat Cu(111) and stepped Cu(*n* + 1, *n*, *n*)–H_2_O interfaces (1 ≤ *n* ≤ 3) and elucidate the effect of different step densities on the structure of interfacial water. Beginning with reference datasets for bulk water and Cu, we employ an AL procedure to efficiently construct a dataset and a transferable MLFF for the Cu–H_2_O interfaces. We show how the use of spatially resolved uncertainties^21^ allows to finely resolve the quality of model predictions in the different regions of interface structures. MD simulations are then conducted to obtain atomically resolved structural properties of the H_2_O network in the contact layer at the various Cu–H_2_O interfaces. A data-driven classification of the local geometries reveals four distinct types of Cu atom environments at the interface. Notably, the Cu(433)–H_2_O interface can be identified as the cross-over where the local structure characteristics of the flat Cu(111) surface can be recovered on a stepped surface.

## 2 Computational details

DFT calculations for the initial Cu database were carried out using VASP version 6.2.0,^22^ with the RPBE functional being used to model the XC contributions to the total energy.^23^ The default PAW setups provided with VASP with a core radius of 2.3 Å for Cu were used. The plane wave energy cutoff was set to 400 eV and the second order Methfessel–Paxton smearing scheme was employed with a smearing width of 0.05 eV. The Brillouin zone was sampled with *k*-point densities corresponding to a 11 × 11 × 11 *Γ*-centered mesh for the one-atom primitive unit cell of fcc-Cu. For surface calculations, only a single *k*-point was considered in the surface normal direction.

Calculations for water were run using VASP version 6.4.2. Due to the short bond lengths in water the hard PAW setups provided with VASP were used, the core radii being 0.8 Å for hydrogen and 1.1 Å for oxygen. The energy cutoff was set to 850 eV, the width for Gaussian smearing to 0.05 Å and only the *Γ*-point of the Brillouin zone was sampled. To account for van-der-Waals interactions, D3 corrections^24^ were computed using the zero damping scheme following previously reported results.^25,26^

Energies and forces for Cu–H_2_O interface structures were calculated using VASP version 6.4.2 with the calculation parameters for bulk water as described above, VASP default Cu PAW setups and the *k*-point grid being taken corresponding to the *k*-point grid for Cu. Only a single *k*-point was considered in the surface normal direction. To approximate the screening of D3 interactions by the metal, only the water molecules and the top Cu layers were included in the evaluation of the D3 contributions to the total energy.^27–29^

MD simulations were carried out using MACE version 0.3.5^30^ models to calculate energies and forces and LAMMPS version 2023.3.28 as the simulation engine.^31^ MACE models were trained using the package as provided with hyperparameters set as described in the following. Models were constructed using a cutoff radius of 5 Å, two layers with rank zero even parity and rank one odd parity hidden features of size 64 each, and a maximum radial order of *l*_max_ = 2. Radial features were constructed using eight Bessel functions and a polynomial cutoff of order *p* = 5. Messages were generated using a MLP with three layers of 64 nodes each and SiLU as the non-linear transfer function. The readouts were performed using a single-layer MLP with 16 nodes. Trainings were run using the AMSGrad optimizer^32^ with hyperparameters and learning rates as given by the defaults provided with the MACE package. First, model parameters were optimized with energy and force weights of 1.0 and 100.0 respectively for a maximum of 1200 epochs with an early stopping patience of 50 epochs. Subsequently, energy and force weights were set to 1000.0 and 100.0 respectively and another maximum of 400 epochs were performed.

The timestep for MD simulations was set to 0.5 fs for all runs, the temperature set to 300 K using a Nosé–Hoover thermostat for simulations in the NVT and NPT ensembles and the pressure set to 1 bar using a Nosé–Hoover barostat for simulations in the NPT ensemble. The characteristic time scales were set to 50 fs for the thermostat and 500 fs for the barostat. The barostat was only coupled to the surface normal direction to not artificially strain the slabs in the directions parallel to the surface.

MD simulations for structural investigations were run using a MACE model trained on the dataset obtained after the AL cycles for Cu–H_2_O interfaces. Interfaces were set up with an initial target water film diameter of 40 Å at a density of 1.0 g cm^−3^. After performing an energy minimization of the initial system and a 10 ps equilibration run in the NVT ensemble, a 200 ps simulation in the NPT ensemble was run. The equilibrium density was then determined from the last 100 ps of the NPT trajectory the same way as was done during the AL iterations. After setting up a new initial system at the equilibrium density and a subsequent energy minimization, 4 ns were simulated in the NVT ensemble. The Cu(111)–H_2_O interface was modelled using a 12 × 12 Cu(111) slab and 1104 water molecules. For the Cu(211)–H_2_O interface, a 12 × 4 Cu(211) slab and 1088 water molecules were used. The Cu(322)–H_2_O interface was represented using a 12 × 3 Cu(322) slab and 1251 water molecules. Finally, a 12 × 2 Cu(433) slab and 1185 water molecules were used to model the Cu(433)–H_2_O interface. The step densities are 1.57 nm^−1^, 0.93 nm^−1^, and 0.66 nm^−1^ for the Cu(211), Cu(322) and Cu(433) slabs respectively.

## 3 Reference databases

Active learning runs for Cu–H_2_O interfaces were initiated based on the combination of three reference databases: one for bulk Cu and Cu slabs in vacuum, one for bulk water and one containing a small number of naively set up Cu(111)–H_2_O interfaces. The generation process for each of these databases is described in this section.

To start the generation of bulk Cu data, the lattice parameter was determined by relaxing the primitive unit cell of fcc Cu. This yielded a value of 3.67 Å, which lies above the experimental lattice parameter of 3.61 Å.^33^ An overestimation compared to the experimental lattice parameter of Cu using the RPBE functional has been reported before.^34^ Subsequently, rattled structures of a 2 × 2 × 2 bulk supercell were generated following the procedure reported in ref. 35 and assuming a Debye temperature of 343 K for Cu. In total, 400 structures were generated, 200 for each temperature of 500 K and 1000 K. To provide information about structures with a non-optimized lattice parameter, bulk structures based on 4 × 4 × 4 supercells with scaled lattice parameters were added to the database: Five structures rattled at a temperature of 500 K were added for each scaling in the range of {−5.0, −2.5, 2.5, 5.0} % of the optimal lattice parameter.

The addition of Cu surface slabs to the database was performed in steps based on the maximum Miller index (MMI) determining the surface orientations. In the first step, only (111), (110) and (100) slabs were included. Bulk-terminated slabs for all symmetrically distinct combinations of orientations were generated based on the relaxed lattice parameter of bulk Cu using the slab generation algorithm implemented in pymatgen.^36,37^ The number of layers were chosen such that a minimum slab thickness of 10 Å was achieved and a vacuum of 10 Å was added in surface normal direction. Slabs were relaxed by performing a geometry optimization starting from the bulk-terminated positions while keeping the unit cell fixed. Both the bulk-terminated slabs and slabs with relaxed atomic positions were added to the database. Additionally, as a starting point for active learning, slabs with MMI one perturbed by random displacements drawn from a normal distributions with standard deviations 0.03 Å, 0.05 Å and 0.10 Å respectively were added. Two sets of ten structures per surface orientation and standard deviation were generated. In the first set, only the outer layers were perturbed. In the second set, displacements were added to all atoms. In total this results in 180 structures obtained by adding random displacements. Further structures were added by adopting the active learning approach based on adversarial loss maximizations.^35,38^ Ensembles of ten NeuralIL^39^ models with a three layer ResNet^40^ core structure with widths [128, 64, 32], a cutoff radius of 4.0 Å, *n*_max_ = 5 and a two dimensional embedding for the atom types were used at each iteration. In a first batch, adversarial loss maximizations with initial displacements drawn from a Gaussian with zero mean and standard distribution of 0.1 Å were performed for 50 replicas each of 1 × 1 and 2 × 2 slabs with the orientations mentioned above. In a second iteration, adversarial loss maximizations were performed with initial displacements drawn with a standard distribution of 0.2 Å. DFT calculations were run for all structures obtained from the optimization procedures and the configurations added to the database. The same active learning procedure described for MMI one surfaces was then repeated for surfaces with orientations with a MMI of two, these being (210), (211) and (221). For surfaces with a MMI of three, adversarial loss maximizations were only performed with initial displacements drawn with a standard distribution of 0.1 Å.

After discarding non-converged calculations, a database consisting of a total of 2276 structures covering bulk Cu and surfaces with symmetrically distinct orientations up to a MMI of three was obtained. As will be discussed below, AL procedures for interfaces were run using MACE, which often requires significantly less training data than NeuralIL. In order to efficiently train neural-network models it is desirable to only use as many data points as necessary to accurately model the region of interest of the potential energy hypersurface. To reduce the total amount of data points in the database, structures were randomly sampled from each of the individual batches to obtain a smaller dataset: 25 structures each from the randomly displaced 2 × 2 × 2 bulk supercells (total 50), rattled 4 × 4 × 4 bulk supercells at non-equilibrium volumes (total 20), bulk-terminated and relaxed slab structures for all symmetrically distinct surface orientations (total 26), MMI one slabs with random perturbations with standard deviation 0.05 Å (total 30), 15 structures for each orientations from adversarial loss optimizations for surfaces with MMI one and MMI two and 10 structures from adversarial loss optimizations for surfaces with MMI three (total 160). After subsampling, a database containing a total of 286 data points was obtained. Note that this database was generated using NeuralIL models and further used to train MACE models, which has been shown to be justified due to the good correlation of uncertainties based on NeuralIL and MACE ensembles.^41^

To make use of the work that has already gone into the construction of databases for water, we chose the 1593 structures of 64 water molecules each published by Cheng *et al.*, originally computed at the revPBE0-D3 level of theory.^42^ The set contains five duplicate structures in the sense of having identical atomic positions, which were removed. Energies and forces were then recomputed for the remaining 1588 structures at the RPBE-D3 level of theory.

In addition to the Cu and water databases, a database of ten Cu(111)–H_2_O interface structures was created by packing water molecules above a 4 × 4 Cu(111) slab at a density of 1.0 g cm^−3^ using GROMACS version 2024.1.^43^ The number of inserted water molecules was chosen such that the density matched the prescribed value and the height of the water film is as close as possible to 20 Å. Such generated configurations each contain 263 atoms in total. A gap of 1.4 Å was assumed for the distance between the outer Cu layer and the region for which the water density, and thus the exact cell height and number of water molecules to insert, was calculated.

## 4 Active learning for copper–water interfaces

A reference dataset with a total of 1884 structures was assembled by combining the Cu, bulk water and Cu(111)–H_2_O databases. Energies and forces for the Cu database were recomputed with the VASP setup used for Cu–H_2_O interfaces to avoid systematic errors arising from different DFT calculation parameters. The reference database served as a starting point for a MD-based AL cycle. At each iteration the dataset was split 90 : 10 into training and validation sets, and an ensemble of five MACE models was trained.

The AL cycle started from a 4 × 4 bulk-terminated Cu(111) slab consisting of five layers. Water molecules were added above the slab such that the bulk water density equaled 1.0 g cm^−3^ and the initial water film diameter was 20 Å. This resulted in the addition of 64 water molecules, yielding structures with 272 atoms in the unit cell. To find the equilibrium density at a given iteration, a 200 ps NPT run was performed after an energy minimization of the initial configuration and a 10 ps equilibration run in the NVT ensemble. The equilibrium volume was then calculated as the mean volume of every 50th frame in the last 100 ps of the NPT simulation run. Subsequently, five new initial configurations with the box volume set to the equilibrium volume were set up. After an initial energy minimization, NVT simulations were run for 200 ps for each of the replicas.

**Fig. 1:**
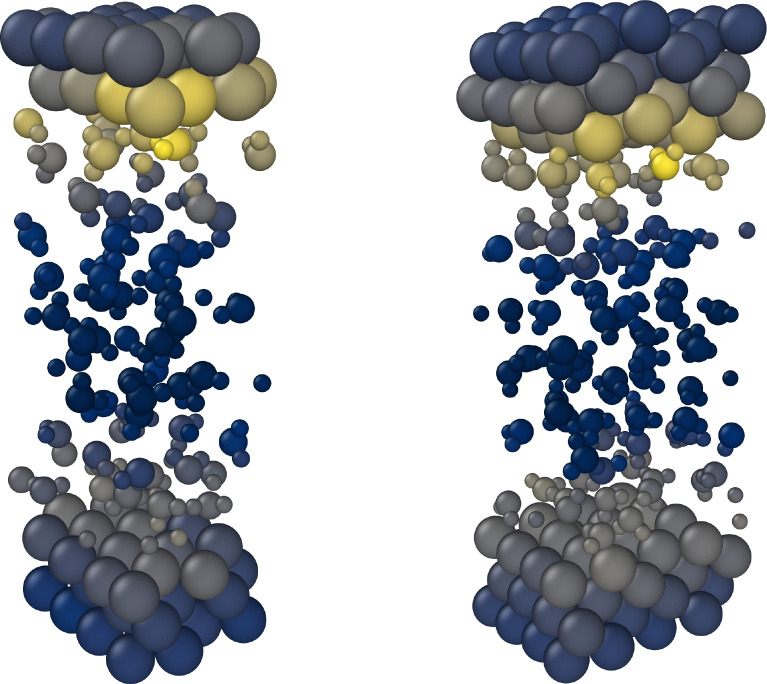
Spatially resolved uncertainties for a Cu(111)–H_2_O (left) and a Cu(211)–H_2_O interface structure (right) selected from NVT trajectories in iterations 3 and 6. A color gradient from dark blue to yellow indicates the lowest and highest local uncertainties respectively. Separate color scales are used for the individual structures.

To avoid sampling correlated frames each NPT and NVT trajectory was divided into two 100 ps segments resulting in 12 segments total. One configuration was selected from each segment by determining the frame featuring the highest locally aggregated force uncertainty.^21^ While the use of structure-wide aggregation is common practice,^20,35,44–46^ it can fail to identify sub-regions featuring high-error.^41^ This has recently been addressed by aggregating only within a defined cutoff radius around each atom. Thereby local atomic uncertainties that still correlate with the actual error are obtained.^21^ We thus calculate local uncertainties by aggregating atomic uncertainties in a neighborhood *N*_*i*_ = {*j* ∈ *N*|||**r**_*i*_ − **r**_*j*_||_2_ < *r*_agg_} of each atom *i* with aggregation cutoff radius *r*_agg_ (*N* denotes the set of all atoms).1
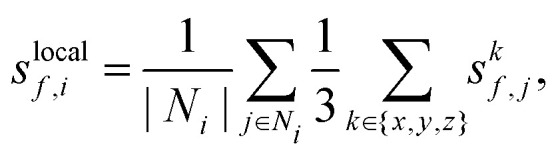
where *s*^*k*^_*f*,*j*_ denotes the uncertainty of a single force component as obtained from the committee and *k* refers to the Cartesian axis. For the present study the aggregation cutoff radius was set to 4 Å. Since both bulk copper and bulk water are already well represented in the initial dataset, uncertainties, and by proxy errors, are expected to be highest at the Cu–H_2_O interface. As visualized in Fig. 1, this is resolved well by the local uncertainties.

**Fig. 2:**
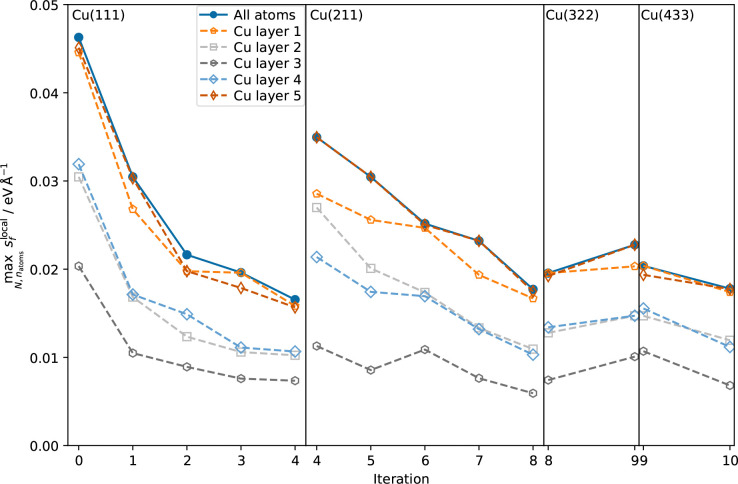
Maximum local force uncertainties observed for the NPT trajectories at each active learning iteration. The solid curves represent the maximum over all atoms, including the water molecules. The dashed curves show the maximum local uncertainty of the Cu atoms resolved layer-wise (inner layer index 3, sublayers indices 2 and 4, interfacial layers indices 1 and 5).

An AL procedure for a given interface was considered converged if the maximum local force uncertainty observed for all atoms and all timesteps in a 200 ps NPT trajectory was smaller than 0.02 eV Å^−1^. According to this criterion, four AL iterations were performed at a temperature of 300 K, Fig. 2, resulting in 48 Cu(111)–H_2_O interface structures being added to the database. After the AL iterations for the Cu(111)–H_2_O interface were completed, the same procedure as detailed above was repeated for the Cu(211)–H_2_O interface. This interface was modelled using a 4 × 2 Cu(211) slab and 86 water molecules. Again, four AL cycles were needed to reach the convergence criterion, resulting in 48 Cu(211)–H_2_O structures that were added to the database. The Cu(322)–H_2_O interface was represented using a 4 × 1 Cu(322) slab and 72 water molecules. A 4 × 1 Cu(433) slab and 92 water molecules were used to model the Cu(433)–H_2_O interface for AL. As will be discussed below, the observed uncertainties for the Cu(322)–H_2_O and Cu(433)–H_2_O systems were already low in the first AL iteration, suggesting that adding additional reference data for these structures can be omitted. However, in the present study we added twelve data points from the first AL cycle for the Cu(322)–H_2_O interface after completing the Cu(211)–H_2_O cycles. Similarly, twelve Cu(433)–H_2_O structures obtained from one AL cycle were added after finishing the procedure for the Cu(322)–H_2_O interface. In total this yielded 120 interface structures that were added to the database over ten subsequent AL cycles. Training a MACE model on this database yields an RMSE of 0.76 meV atom^−1^ for the energies and 20.42 meV Å^−1^ for the forces when evaluating errors on the whole training set.

In all iterations the highest uncertainties are observed directly at the interface, while both in the bulk Cu and bulk H_2_O regions, uncertainties are low, see Fig. 1. The difference in uncertainties for the interface and bulk regions is further apparent in Fig. 2. At all AL iterations the atomic uncertainties are highest for an outer layer Cu atom or an atom in a water molecule in the contact layer. Since the initial database contains no structures sampled from MD trajectories, high maximum local uncertainties are observed when starting the AL process. After converging for the Cu(111)–H_2_O interface, a jump is observed when moving on to the Cu(211) surface, as no data for stepped interfaces is yet present in the database. However, even for the first Cu(211) iteration, uncertainties for Cu atoms in the bulk layer are already low and no systematic reduction is observed in further AL iterations. The databases used to train the models used in the AL iterations for Cu(322)–H_2_O and Cu(433)–H_2_O interfaces did not contain any training structures of the respective interfaces they were applied on. Still, uncertainties already satisfy the specified cutoff criterion in the first iteration on the respective surfaces. A small increase local force uncertainty is observed for the Cu(322)–H_2_O interface after adding data, but was not investigated further and no additional AL cycles were run.

## 5 Structure at the interface

**Fig. 3:**
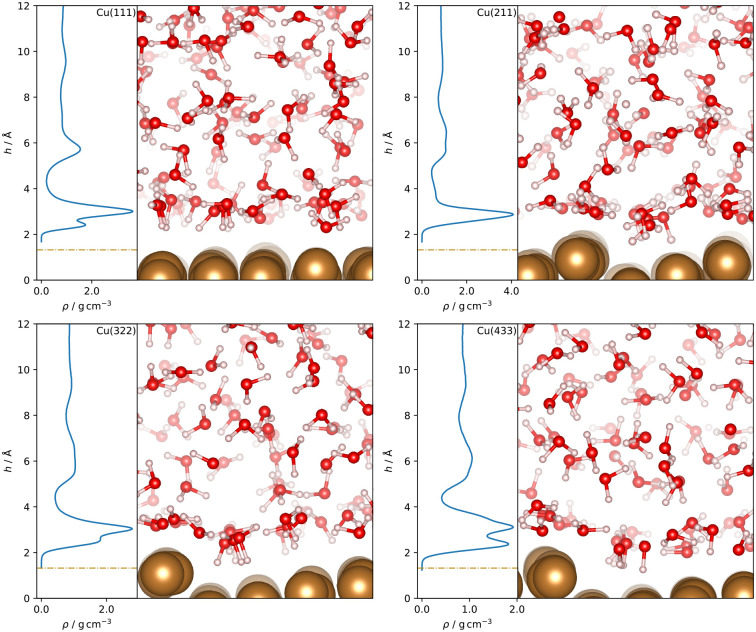
Water density profiles as a function of the distance *h* from the topmost surface layer for Cu–H_2_O interfaces with different Cu surface orientations. The density profiles were obtained by averaging the distances of the centers of mass of the water molecules from the surface over the trajectory after discarding the first 10 ps. The position *h* = 0 Å of the topmost surface layer in surface normal direction was computed as the mean *z*-position of all Cu atoms in the top layer at each timestep. Visualized frames were obtained from the trajectories by selecting the snapshot featuring the lowest instantaneous hydrogen distance from the surface.

Water density profiles were obtained from the 4 ns MD simulations of Cu–H_2_O interfaces using the MACE model trained on the final dataset. They are shown in Fig. 3, along with snapshots of representative structures of the water contact layer. The Cu(111)–H_2_O interface exhibits a distinct double-peak structure with maxima of 3.62 g cm^−3^ and 1.75 g cm^−3^ around 2.99 Å and 2.41 Å above the outermost Cu layer, in agreement with previous studies.^10,11^ A similar ordering is also found for *e.g.* Pt(111)^11–16^ but not for the more noble Au(111).^11,15,16^ The tendency towards ordering of interfacial water molecules within the water contact layer indicates chemisorbed water. This is illustrated in the snapshot in Fig. 3 where a water molecule at approximately 2.4 Å with the oxygen atom oriented towards the Cu slab.

**Fig. 4:**
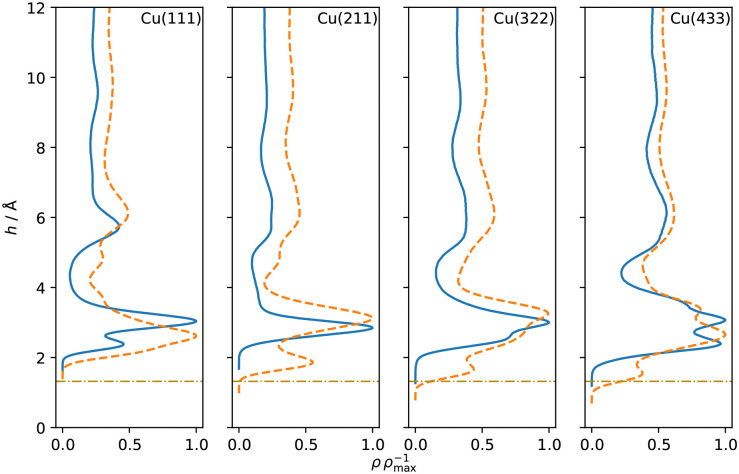
Density profiles of oxygen (solid blue) and hydrogen (dashed orange) as a function of the distance *h* from the topmost surface layer for Cu–H_2_O interfaces with different Cu surface orientations. The curves were normalized with respect to their global maximum. Dash-dotted lines indicate the covalent radius of Cu.

**Fig. 5:**
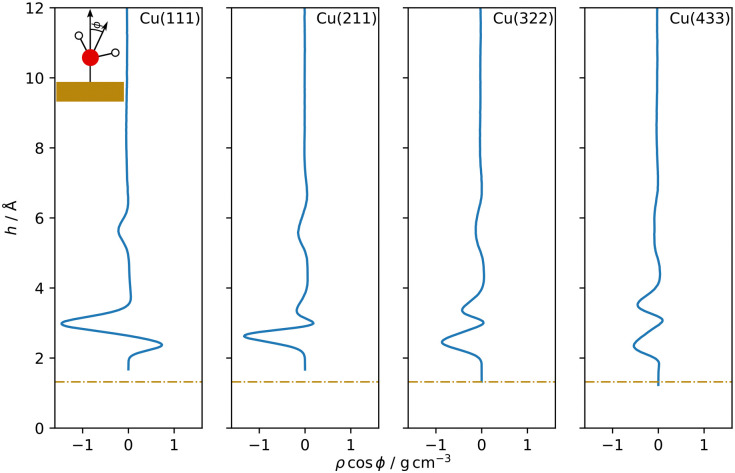
Dipole orientation distribution profiles as a function of the distance *h* from the topmost surface layer for Cu–H_2_O interfaces with different Cu surface orientations. Cos *ϕ* is the cosine of the angle between the dipole vector, calculated as the bisector of the water molecule orientated from the oxygen to the mid point of the two hydrogen atoms, and the surface normal (see insert). Dash-dotted lines indicate the covalent radius of Cu.

Separating the density profiles for the individual atom types, as shown in Fig. 4, also points towards the first density maximum representing chemisorbed water, as only a single peak is observed for the hydrogen atoms. This peak represents both chemisorbed water molecules, with hydrogen atoms pointing away from the surface, and water molecules oriented with the hydrogen atoms towards the surface, which are mapped to the global maximum of the density curve for water, Fig. 3. This is additionally evident from the density profiles weighted with cos *ϕ*, the cosine of the angle between the dipole vector and the surface normal, as visualized in Fig. 5. A positive first peak shows the water molecules closest to the slab to be oriented primarily with the oxygen towards the surface, while the negative peak at 2.99 Å indicates the opposite orientation for the corresponding water molecules. This is also similar to the Pt(111)–H_2_O interface, for which a compensation of the dipoles of chemisorbed water by the outer layer is reported.^14^

A double peak structure is not observed for the Cu(211)–H_2_O interface, where only a strong single peak is present at a distance of 2.86 Å from the surface. Qualitatively, this is understood as a consequence of the step edge combined with a short plateau, since water molecules primarily adsorb on the ridge atoms due to their lower coordination. As illustrated by the Cu(211)–H_2_O interface snapshot in Fig. 3, water primarily adsorbing at the ridge sites induces a strict H-down orientation of water molecules at the adjacent crevice to facilitate hydrogen bonding to the molecules adsorbed at the ridge sites. This is reflected in the density profile for hydrogen (Fig. 4), which now features two peaks in contrast to the Cu(111)–H_2_O interface. The first of these maxima corresponds to water molecules at the step crevices. For all stepped interfaces, hydrogen densities are non-zero in the region bounded by the covalent radius of Cu, which is a consequence of both the definition of the reference height as the instantaneous mean of the heights of the Cu atoms in the top layer in surface normal direction and the aforementioned arrangement around the step. The local structure at the steps furthermore explains the negative first peak in the angular weighted density profiles for all stepped interfaces (Fig. 5). In contrast to results reported for the Pt(211)–H_2_O interface,^14^ the Cu(211)–H_2_O interface exhibits a small positive peak, attributed to the water molecules at the ridge sites.

Decreasing the step density leads to the formation of a second peak in the contact layer, Fig. 3. Specifically, a weak shoulder at the Cu(322)–H_2_O interface and a distinct second peak at the Cu(433)–H_2_O interface are observed. However, the origin of the two peaks is different than for the flat Cu(111) surface, since the structure is still strongly influenced by the undercoordinated ridge sites. The depth of the first minimum in the cosine-weighted density curves, Fig. 5, is lowered with increasing step density. As can be seen in the snapshot of the Cu(433)–H_2_O interface in Fig. 3 and will be discussed in more detail below, a sufficiently long plateau allows for water molecules to orient with the oxygen towards the surface, similar as at the Cu(111)–H_2_O interface.

**Fig. 6:**
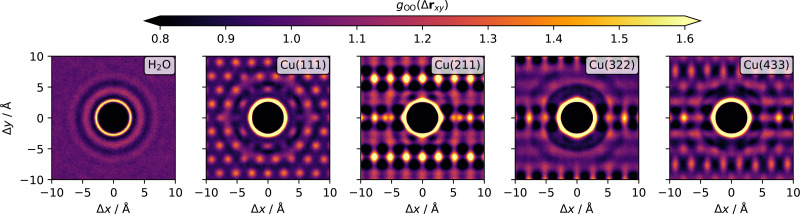
2D oxygen–oxygen pair correlation functions for bulk water or water in the contact layers of the interfaces respectively. The maximum distance h defining the contact layer was chosen as the local minimum of the density profiles (Fig. 3) following the the global maximum. The 2D-PCF for bulk water is computed based on a slab with thickness 5 Å in *z*-direction.

Density curves such as those shown in Fig. 3–5 can also be obtained by AIMD.^11,15^ The comparatively longer time-scales that become accessible through MLFF-backed MD make it possible to obtain reliable statics for local resolution. Here we investigate interfacial water structure in the surface parallel directions by calculating the oxygen–oxygen 2D pair correlation functions (2D PCF) as given by^16,47^2
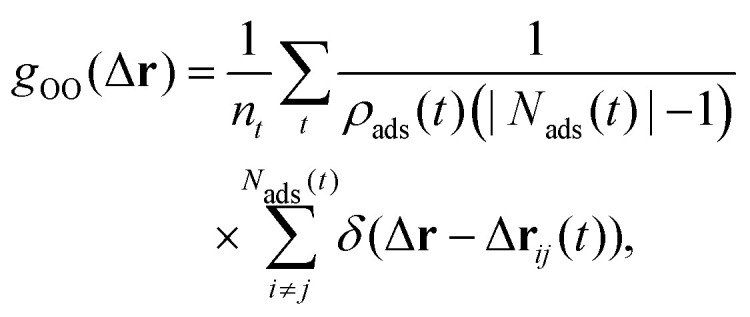
where Δ**r**_*ij*_ is a two-dimensional vector that denotes the pairwise distance in the directions parallel to the surface, *ρ*_ads_(*t*) the surface number density of oxygen atoms in the contact layer at time *t*, *N*_ads_(*t*) the set of oxygen atoms in the contact layer at time *t* and *n*_*t*_ the total number of timesteps. The 2D PCFs for both bulk water and the different interfaces are visualized in Fig. 6. On the flat Cu(111) surface, a similar structure as for the Pt(111) surface is found.^16^ A ring of large *g*_OO_(Δ**r**) values indicating the first solvation shell is observed, followed by weaker peaks arising from the second and third solvation shells. The 2D PCF does however not converge to a constant value of 1.0 with increasing distance, as is the asymptotic limit for of bulk water, but exhibits peaks induced by the underlying Cu atoms. Notably, the third solvation shell matches the distance of 6.86 Å of the fourth nearest in-plane neighbor of a Cu atom in the top layer and therefore shows pronounced peaks at these locations. As evident from Panel Q of Fig. 1 in ref. 16, this is in contrast to Pt, for which such a lattice parameter match is not observed. The introduction of a step on the surface leads to pronounced changes in the shapes of the 2D PCFs. On the Cu(211) surface, which features the highest step density of all investigated surfaces, a highly anisotropic profile is observed. The 2D PCF is mainly dominated by the steps, even in the short-range regime, with also the first solvation shell being distorted. Strong peaks are observed at the ridge Cu atoms even at longer distances indicating the steps as the preferred adsorption sites. Anisotropic profiles are also observed for the Cu(322)–H_2_O and Cu(433)–H_2_O interfaces, but the effects are less pronounced due to the decreasing step densities. The 2D oxygen–oxygen PCF for the water molecules in the contact layer on the Cu(433) surface already shows that the pattern observed on the flat Cu(111) surface is partially recovered due the longer (111)-like plateau of the Cu(433) surface.

**Fig. 7:**
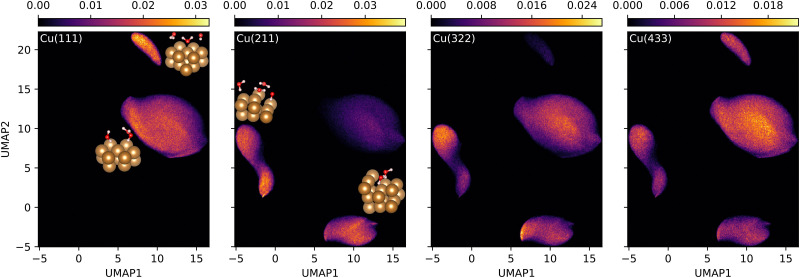
Two-dimensional density histograms of the UMAP representations of the spherical Bessel descriptors of the top layer Cu environments. A single UMAP has been computed using 40 neighbors, a minimum distance of 0.2 and the Euclidean distance metric for the whole set of data and was subsequently split into subsets representing the individual interfaces, for which individual histograms were calculated. Representative environments taken from snapshots illustrate the types of substructures constituting the individual clusters.

To illustrate how decreasing the step density recovers structural features observed for the flat Cu(111)–H_2_O interface we investigate the local environments of the atoms in the contact layer sampled during the MD simulations. Using spherical Bessel descriptors^39,48^ to represent the local environments of Cu atoms in the top layer, we encode the local environment in a rotationally invariant manner. Snapshots were taken at 1 ps intervals from the simulations for each interface. The combined set of descriptors is visualied using the dimensionality reduction technique UMAP^49^ in Fig. 7.

At the Cu(111)–H_2_O interface, two distinct types of local Cu environments are observed, corresponding to the two types of water orientations producing the opposite peaks observed in Fig. 5. The smaller patch in Fig. 7 corresponds to chemisorbed water with hydrogen atoms oriented away from the surface (positive cos *ϕ*) and shorter Cu–O distances. The larger patch corresponds to environments where the hydrogen atoms are oriented towards the surface. Environments in the latter region are also observed at the Cu(211)–H_2_O interface. On Cu(211), chemisorption primarily takes place at the undercoordinated ridge sites, while water molecules in the crevice of the step are almost exclusively oriented with the hydrogen atoms oriented towards the surface (Fig. 3), forming distinct UMAP patches, Fig. 7. The high step density of the Cu(211) surface geometrically restricts interfacial water molecules from orienting in a (111)-like chemisorption geometry. This results in the absence of the corresponding UMAP patch, in agreement with the behavior observed in Fig. 3–5. Reducing the step density exposes longer plateaus, thereby alleviating these restrictions and making Cu(111)-like chemisorption geometries possible, as seen for the Cu(322) and Cu(433) panels of Fig. 7. The Cu(322) surface, featuring a shorter plateau than Cu(433), exhibits a sparser population of the Cu(111)-like chemisorption patch. The Cu(433) surface clearly shows this, consistent with the oxygen atoms oriented towards the surface in the corresponding snapshot Fig. 3.

Interestingly, no new regions of configuration space are visited during the simulations of the Cu(322)–H_2_O and Cu(433)–H_2_O interfaces compared to Cu(111)–H_2_O and Cu(211)–H_2_O. This is in accordance with the already low uncertainties in the first AL cycles on the higher-index surfaces, Fig. 2. We therefore also expect interfaces to less idealized surfaces, which can be conceptualized as being composed of individual elements present in simpler model systems, to be predicted well by an MLFF trained only on the these model interfaces.

**Fig. 8:**
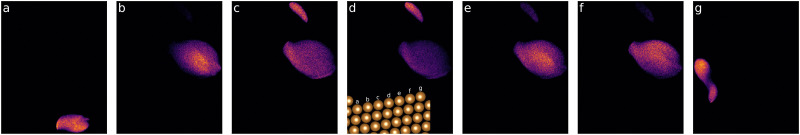
Two-dimensional density histogram of the UMAP representations of the spherical Bessel descriptors of the top layer Cu atom environments of the Cu(433)–H_2_O interface. Panels (a–g) visualize the environments observed at the individual rows, which are enumerated as shown in the inset of panel d. The individual histograms sum to the values visualized in Fig. 7, individual color scales were used for visibility.

A more fine-grained analysis of the environments is achieved by separating the data according to rows parallel to the step edge, as shown for the Cu(433)–H_2_O interface are in Fig. 8. The environments of the crevice and ridge Cu atoms are visualized in panels a and g respectively and form two separate clusters of environment types as discussed above. Due to the geometric restriction of the water molecules in the direct vicinity of the step, where the ridge atoms are the preferred sites for chemisorption, no environments indicating (111)-like chemisorption geometries are found for Cu atoms in the rows adjacent to the crevice and ridge rows (panels b and f respectively). As discussed in the context of Fig. 6, the interfacial structure of water approaches that of the Cu(111)–H_2_O interface for high-index Cu(*n* + 1, *n*, *n*) surfaces. This is further supported by the environments of the top layer Cu atoms located in rows further from the step being clustered similarly to those of the Cu(111)–H_2_O interface, as evident in panels c–e in Fig. 8.

Characterizing the local structure of water is a long-standing challenge, with different local order parameters often giving ambiguous results.^50,51^ The UMAP analysis highlights how a data-driven approach based on local invariant descriptors, enables a clear classification of the 32 million geometries sampled during the MD simulations without relying on, potentially biased, a-priori intuition of which geometric features to probe.

## Conclusions

In the present study we have demonstrated that high step densities lead to a fundamentally different structuring of interfacial water compared to the ideal, flat surface and that lowering the step density gradually recovers behavior similar to the interface to the flat surface. We recover previously reported results for the Cu(111)–H_2_O interface, such as the double peak structure in the density and the Cu ontop sites being preferred. On all stepped surfaces, we observe chemisorption taking place mainly on the step sites, as opposed to the terrace sites. Further, we connect observed results and a data-driven approach to identify common types of atomic environments across the differently oriented surfaces. In order to obtain these results we both make use of existing databases and also developed a locally sensitive active learning workflow for Cu–H_2_O interfaces that makes use of the high data efficiency of equivariant MLFFs. This minimizes the amount of computationally demanding reference calculations, in this case even removing the need for AIMD trajectories entirely. The workflow allows for systematic extension of the database to investigate a wider range of systems. By identifying the relevant local environments and training on according model systems, we conceive this approach to be suitable for modelling interfaces to less idealized surfaces. In order to achieve more realistic models, some aspects remain to be addressed. For the present system in particular, including different oxidization states of copper is essential to move towards a more realistic system.^52,53^ Furthermore, no water dissociation is observed in the present study and no dissociated species are considered. These aspects can already be tackled with currently available MLFFs,^54^ but may require the use of enhanced sampling techniques to be simulated efficiently.^55^ Additionally, the treatment of long-range interactions and the inclusion of electric fields in MLFFs is still an open research question and obviously of vital importance for modelling electrochemical interfaces.

## Author contributions

Johannes Schörghuber: conceptualization, methodology, software, validation, investigation, visualization, writing – original draft Nina Bučková: Investigation Esther Heid: conceptualization, methodology, supervision, writing – review & editing Georg K. H. Madsen: conceptualization, methodology, supervision, writing – review & editing, project administration.

## Data availability

The generated dataset, models, input files, initial structures for MD simulation runs, and a Jupyter notebook for the presented trajectory analysis are available on Zenodo at https://doi.org/10.5281/zenodo.14755563.

## Conflicts of interest

There are no conflicts to declare.

## References

[cit1] Björneholm O., Hansen M. H., Hodgson A., Liu L.-M., Limmer D. T., Michaelides A., Pedevilla P., Rossmeisl J., Shen H., Tocci G., Tyrode E., Walz M.-M., Werner J., Bluhm H. (2016). Chem. Rev..

[cit2] Seh Z. W., Kibsgaard J., Dickens C. F., Chorkendorff I., Nørskov J. K., Jaramillo T. F. (2017). Science.

[cit3] Hori Y., Takahashi I., Koga O., Hoshi N. (2002). J. Phys. Chem. B.

[cit4] Hori Y., Takahashi I., Koga O., Hoshi N. (2003). J. Mol. Catal. A: Chem..

[cit5] Nitopi S., Bertheussen E., Scott S. B., Liu X., Engstfeld A. K., Horch S., Seger B., Stephens I. E. L., Chan K., Hahn C., Nørskov J. K., Jaramillo T. F., Chorkendorff I. (2019). Chem. Rev..

[cit6] Auer A., Sarabia F. J., Winkler D., Griesser C., Climent V., Feliu J. M., Kunze-Liebhäuser J. (2021). ACS Catal..

[cit7] Winkler D., Leitner M., Auer A., Kunze-Liebhäuser J. (2024). ACS Catal..

[cit8] Carrasco J., Hodgson A., Michaelides A. (2012). Nat. Mater..

[cit9] Groß A., Sakong S. (2022). Chem. Rev..

[cit10] Natarajan S. K., Behler J. (2016). Phys. Chem. Chem. Phys..

[cit11] Heenen H. H., Gauthier J. A., Kristoffersen H. H., Ludwig T., Chan K. (2020). J. Chem. Phys..

[cit12] Lan J., Rybkin V. V., Iannuzzi M. (2020). J. Phys. Chem. Lett..

[cit13] Mikkelsen A. E. G., Schiøtz J., Vegge T., Jacobsen K. W. (2021). J. Chem. Phys..

[cit14] Chen A., Le J.-B., Kuang Y., Cheng J. (2022). J. Chem. Phys..

[cit15] Liu S., Vijay S., Xu M., Cao A., Prats H., Kastlunger G., Heenen H. H., Govindarajan N. (2023). J. Chem. Phys..

[cit16] Gäding J., Della Balda V., Lan J., Konrad J., Iannuzzi M., Meißner R. H., Tocci G. (2024). Proc. Natl. Acad. Sci. U. S. A..

[cit17] Natarajan S. K., Behler J. (2017). J. Phys. Chem. C.

[cit18] Arrigo R. (2022). Curr. Opin. Green Sustainable Chem..

[cit19] Smith J. S., Nebgen B., Lubbers N., Isayev O., Roitberg A. E. (2018). J. Chem. Phys..

[cit20] Piaggi P. M., Selloni A., Panagiotopoulos A. Z., Car R., Debenedetti P. G. (2024). Faraday Discuss..

[cit21] Heid E., Schörghuber J., Wanzenböck R., Madsen G. K. H. (2024). J. Chem. Inf. Model..

[cit22] Kresse G., Furthmüller J. (1996). Phys. Rev. B: Condens. Matter Mater. Phys..

[cit23] Hammer B., Hansen L. B., Nørskov J. K. (1999). Phys. Rev. B: Condens. Matter Mater. Phys..

[cit24] Grimme S., Antony J., Ehrlich S., Krieg H. (2010). J. Chem. Phys..

[cit25] Morawietz T., Singraber A., Dellago C., Behler J. (2016). Proc. Natl. Acad. Sci. U. S. A..

[cit26] Montero de Hijes P., Dellago C., Jinnouchi R., Schmiedmayer B., Kresse G. (2024). J. Chem. Phys..

[cit27] McNellis E. R., Meyer J., Reuter K. (2009). Phys. Rev. B: Condens. Matter Mater. Phys..

[cit28] Mercurio G. (2010). Phys. Rev. Lett..

[cit29] Tonigold K., Groß A. (2012). J. Comput. Chem..

[cit30] Batatia I., Kovacs D. P., Simm G., Ortner C., Csányi G. (2022). Adv. Neural. Inf. Process. Syst..

[cit31] Thompson A. P., Aktulga H. M., Berger R., Bolintineanu D. S., Brown W. M., Crozier P. S., in’t Veld P. J., Kohlmeyer A., Moore S. G., Nguyen T. D., Shan R., Stevens M. J., Tranchida J., Trott C., Plimpton S. J. (2022). Comput. Phys. Commun..

[cit32] Reddi S. J. , Kale S. and Kumar S., *arXiv*, 2019, preprint, arXiv:1904.09237 10.48550/arXiv.1904.09237

[cit33] Straumanis M. E., Yu L. S. (1969). Acta Crystallogr., Sect. A.

[cit34] Luo S., Zhao Y., Truhlar D. G. (2012). J. Phys. Chem. Lett..

[cit35] Carrete J., Montes-Campos H., Wanzenböck R., Heid E., Madsen G. K. H. (2023). J. Chem. Phys..

[cit36] Tran R., Xu Z., Radhakrishnan B., Winston D., Sun W., Persson K. A., Ong S. P. (2016). Sci. Data.

[cit37] Ong S. P., Richards W. D., Jain A., Hautier G., Kocher M., Cholia S., Gunter D., Chevrier V. L., Persson K. A., Ceder G. (2013). Comput. Mater. Sci..

[cit38] Schwalbe-Koda D., Tan A. R., Gómez-Bombarelli R. (2021). Nat. Commun..

[cit39] Montes-Campos H., Carrete J., Bichelmaier S., Varela L. M., Madsen G. K. H. (2022). J. Chem. Inf. Model..

[cit40] He K. , Zhang X., Ren S. and Sun J., *arXiv*, 2015, preprint, arXiv:1512.03385 10.48550/arXiv.1512.03385

[cit41] Wanzenböck R., Heid E., Riva M., Franceschi G., Imre A. M., Carrete J., Diebold U., Madsen G. K. H. (2024). Digit. Discovery.

[cit42] Cheng B., Engel E. A., Behler J., Dellago C., Ceriotti M. (2019). Proc. Natl. Acad. Sci. U. S. A..

[cit43] Abraham M. J., Murtola T., Schulz R., Páll S., Smith J. C., Hess B., Lindahl E. (2015). SoftwareX.

[cit44] Kahle L., Zipoli F. (2022). Phys. Rev. E.

[cit45] Jørgensen P. B. , Busk J., Winther O. and Schmidt M. N., *arXiv*, 2023, preprint, arXiv:2312.04174 10.48550/arXiv.2312.04174

[cit46] Kellner M., Ceriotti M. (2024). Mach. Learn.: Sci. Technol..

[cit47] Bernard E. P., Krauth W. (2011). Phys. Rev. Lett..

[cit48] Kocer E., Mason J. K., Erturk H. (2020). AIP Adv..

[cit49] McInnes L., Healy J., Saul N., Großberger L. (2018). J. Open Source Softw..

[cit50] Duboué-Dijon E., Laage D. (2015). J. Phys. Chem. B.

[cit51] Doi H., Takahashi K. Z., Aoyagi T. (2021). J. Chem. Phys..

[cit52] Favaro M., Xiao H., Cheng T., Goddard W. A., Yano J., Crumlin E. J. (2017). Proc. Natl. Acad. Sci. U. S. A..

[cit53] Lee S. H., Lin J. C., Farmand M., Landers A. T., Feaster J. T., Avilés Acosta J. E., Beeman J. W., Ye Y., Yano J., Mehta A., Davis R. C., Jaramillo T. F., Hahn C., Drisdell W. S. (2021). J. Am. Chem. Soc..

[cit54] Quaranta V., Behler J., Hellström M. (2019). J. Phys. Chem. C.

[cit55] Zeng Z., Wodaczek F., Liu K., Stein F., Hutter J., Chen J., Cheng B. (2023). Nat. Commun..

